# Editorial: User Modeling and Recommendations

**DOI:** 10.3389/fdata.2022.923397

**Published:** 2022-05-25

**Authors:** Denis Helic, Ujwal Gadiraju, Marko Tkalcic

**Affiliations:** ^1^Faculty of Computer Science and Biomedical Engineering, Institute of Interactive Systems and Data Science, Graz University of Technology, Graz, Austria; ^2^Web Information Systems, Faculty of Electrical Engineering, Mathematics, and Computer Science, Delft University of Technology, Delft, Netherlands; ^3^HICUP Lab, Faculty of Mathematics, Natural Sciences and Information Technologies, University of Primorska, Koper, Slovenia

**Keywords:** recommender systems, user analysis, user modeling, user behavior, recommendations

## 1. Introduction

The behavior of users in the digital world (e.g., online shopping, social media activity, etc.) is increasingly supported by recommender systems (Ricci et al., 2015). Recommender systems are mainly data-driven, based on behavioral data, such as ratings, likes, purchases or general interaction and consumption (Bell et al., 2007). Although these systems are useful for both users and service providers, they have several drawbacks including the cold start problem (i.e., the data sparsity in the initial stages of system deployment), various biases resulting from biases in the user-generated data (Ntoutsi et al., 2020) (i.e., gender, popularity, or selection bias) or the limited explainability of the data (i.e., using data without understanding the root cause of behaviors). Hence, recent work has started to adopt approaches that include sophisticated user analysis and modeling as well as algorithms that reduce biases (Elahi et al., 2021) and generate fair and explainable recommendations (Zhang and Chen, 2020).

Frequently, these intelligent systems take advantage of psychological models to explain and predict user interactions with the systems (Tkalcic and Chen, 2015), influence user interaction through novel interfaces (Gupta et al., 2022), and allow for a deeper understanding of user behavior (Wölbitsch et al., 2019), including user trust in the systems (Erlei et al., 2020), and their reliance on such systems (Tolmeijer et al., 2021; Erlei et al., 2022), user preferences and needs (Wölbitsch et al., 2020; Najafian et al., 2021), which in turn also allow for more generalizable results. In complement, digital behavior in other systems has also been used to infer user characteristics. For example, social media activities have been used to analyze, model, and predict user behavior in recommender systems (Eberhard et al., 2019).

## 2. Research Topic Content

Given the aforementioned context, this Research Topic encouraged submissions on the usage of user behavior analysis and user models in the broad landscape of recommender systems. In particular, we encouraged the authors to submit original research articles, case studies, reviews, theoretical and critical perspectives, and viewpoint articles on the following topics:

- User analysis and models that explain online behavior
- Methods for Analyzing User Behavior
- Recommender Systems and Algorithms, and
- Algorithmic Fairness and Transparency.

Within this collection we accepted eight original research articles. The author's affiliation countries were diverse, including Europe (Italy, Norway, Germany, UK, the Netherlands, and Austria), North America (USA and Canada), and Asia (Japan).

The topics cover (i) *user behavior and models*, (ii) *recommender systems and algorithms*, (iii) *user analysis and observational studies*, and (iv) *adaptive systems*.

In their work, Akhuseyinoglu and Brusilovsky explored behavioral patterns for data-driven modeling of learners' individual differences by using a large volume of learner data collected in an online practice system. The authors showed that their proposed data-driven model of individual differences outperforms conventional models in predicting learner performance and engagement. Prange and Sonntag synthesized and implemented 170 digital pen features, and evaluated this feature set in paper-pencil-based neurocognitive assessments in the medical domain. The authors showed that the proposed feature set outperforms three conventional approaches for cognitive tests considered in a binary classification task. In their work, Morita et al. developed a browser extension to alleviate negative emotions during web use by leveraging the cognitive architecture Adaptive Control of Thought-Rational (ACT-R) as a model of human memory and emotion. The authors empirically demonstrate that the counterbalanced model suppresses negative ruminative web browsing.

Elahi et al. developed a university recommender system, eliciting user preferences as ratings to build predictive models and generate personalized university ranking lists. Through two studies the authors evaluated which recommender approaches demonstrated the highest predictive value and explored preferred university features. In their work, Zarindast and Wood propose an auto-routine and color scheme recommender system for home-based smart lighting exploiting historical data from users. The authors found that models based on similar users increases the prediction accuracy, with and without prior knowledge about user preferences. Alslaity and Tran presented a study that explored how users with different characteristics get influenced by the various persuasive principles that a recommender system uses, revealing that persuasive principles can enhance user experiences. The authors showed that, among the factors considered in this study, culture, personality traits, and the domain of recommendations have a relatively higher impact on the influence of persuasive principles.

In their work, Sikdar et al. quantified the effects of signaling gender through gender specific user names, on the success of reviews written on the popular amazon.com shopping platform. The authors contrasted the effects of gender signaling and performance on the review helpfulness ratings using matching experiments, and found no general trend that gendered signals or performances influence overall review success, although strong context-specific effects were observed.

Finally, Wilschut et al. present a framework for speech-based word learning using an adaptive model that was developed for and tested with typing-based word learning. The authors demonstrate that typing- and speech-based learning result in similar behavioral patterns that can be used to reliably estimate individual memory processes, and that adaptive learning benefits transfer from typing-based learning, to speech based learning.

## Author Contributions

All authors listed have made a substantial, direct, and intellectual contribution to the work and approved it for publication.

## Conflict of Interest

The authors declare that the research was conducted in the absence of any commercial or financial relationships that could be construed as a potential conflict of interest.

## Publisher's Note

All claims expressed in this article are solely those of the authors and do not necessarily represent those of their affiliated organizations, or those of the publisher, the editors and the reviewers. Any product that may be evaluated in this article, or claim that may be made by its manufacturer, is not guaranteed or endorsed by the publisher.
